# Effects of Metacognitive Strategies on the Self-Regulated Learning Process: The Mediating Effects of Self-Efficacy

**DOI:** 10.3390/bs9120128

**Published:** 2019-11-26

**Authors:** Daisuke Akamatsu, Motoyuki Nakaya, Ryuhei Koizumi

**Affiliations:** 1 Graduate School of Education and Human Development, Nagoya University, Nagoya city, Aichi Prefecture 4660802, Japan; nakaya.motoyuki@b.mbox.nagoya-u.ac.jp; 2 Faculty of Applied Sociology, Kindai University, Higashiosaka City, Osaka Prefecture 577-8502, Japan; koizumi@socio.kindai.ac.jp

**Keywords:** learning strategy, self-efficacy, self-regulated learning

## Abstract

The purpose of this study was to examine the effects of metacognitive strategies on self-regulated learning processes, focusing on the mediating effects of self-efficacy. The surveys were conducted in December 2016 (Time 1) and January 2017 (Time 2). One hundred and five undergraduates enrolled at a Japanese university participated in this survey study, consisting of two surveys conducted one month apart. The questionnaires measured the use of metacognitive strategies (i.e., planning strategy and monitoring strategy), self-efficacy, general learning behaviors (behavioral engagement and persistence), and the use of cognitive strategies (i.e., writing-repetition strategy and deep-processing strategy). First, cross-lagged structure equation modeling revealed that the use of planning strategy enhanced self-efficacy. Second, path analysis examined relationships between metacognitive strategies, general learning behaviors, and cognitive strategies. It revealed that (a) general learning behaviors were promoted by metacognitive strategies mediated by self-efficacy and (b) cognitive strategies were almost directly affected by the monitoring strategy. The current study reveals that general learning behaviors and cognitive strategies involve different processes than metacognitive strategies.

## 1. Introduction

### 1.1. Metacognitive Strategies in Self-Regulated Learning

Past research on self-regulated learning offered a great deal of empirical evidence on students’ autonomous involvement in their own learning processes [1]. Self-regulated learning comprises three major components: motivation, metacognition, and learning strategies. In particular, metacognition plays a crucial role in the execution of self-regulated learning, as it regulates individuals’ cognitive processes and general learning behaviors [2].

Learning strategies by which learners actively regulate their own cognitive processes are conceptualized as metacognitive strategies [3]. They include planning strategy and monitoring strategy. The planning strategy entails, for example, setting learning goals or trying to secure sufficient study time. The monitoring strategy refers, for instance, to the awareness one gains of his/her own understandings of cognitive processes or thinking about how to learn.

### 1.2. The Cognitive Function of Metacognitive Strategies: Promoting Cognitive Strategy Use

Metacognitive strategies are beneficial in several ways. First, they enhance cognitive strategies, which, in turn, directly relate to individuals’ information processing. This assumption is theoretically grounded in the findings of metacognition research, which claim that metacognition promotes individual memory and information processing [4].

Cognitive strategies are different subconstructs of learning strategies than metacognitive strategies [5]. Although they include many substrategies, past research has shown that strategies with deep cognitive-processing (e.g., elaboration, categorization, etc.) improve academic achievement, while strategies with surface cognitive processing (e.g., writing-repetition) do not promote that achievement [6].

It is thus not surprising that published literature has indicated that metacognitive strategies are meant to promote cognitive strategy use. Vermunt focused on the differences of the functions of metacognitive regulation strategies and cognitive-processing strategies. He established the model whereby learners’ orientations promote the use of metacognitive regulation strategies, which in turn affect processing strategy use [7]. A number of the following studies have supported the effects of metacognitive strategy use on cognitive strategy use [8,9,10,11,12].

Although an overwhelming majority of these past studies on the causality between learning strategies treated each metacognitive strategy as a single-factored strategy [8,11], Sato applied two substrategies, the planning strategy and the monitoring strategy, as metacognitive strategies. Sato’s study examined the effects of two metacognitive strategies and showed that the use of the monitoring strategy promoted cognitive strategy use. He posits that the monitoring strategy prompts learners to reflect on their own understanding and learning strategies, which encourages them to focus on their cognitive processes and use cognitive strategies in order to deepen their understanding [10].

### 1.3. The Motivational Function of Metacognitive Strategies: Promoting General Learning Behaviors

In addition to the effects shown thus far, metacognitive strategies have also been shown to enhance general learning behaviors [13,14,15]. As metacognitive strategies involve individuals’ learning processes, they foster general learning behaviors, such as effort, persistence, and behavioral engagement [13,14].

The process by which metacognitive strategies enhance general learning behaviors has been explained by motivational regulation. Metacognitive strategies were conceptualized to be part of motivational regulation strategies [15]. Schwinger and his colleagues examined the effects of motivational regulation strategies, including metacognitive strategies [13,16]. Schwinger and Stiensmeier-Pelster revealed that proximal goal-setting strategy use promotes learning-effort management, which in turn directly contributes to academic achievement [16]. Schwinger and Otterpohl examined the effects of seven motivational regulation strategies on learning effort. Their results showed that the proximal goal-setting strategy (akin to the planning strategy) exerted more influence on learning effort than any other strategy [13]. Thus, their findings consistently demonstrated that proximal goal-setting strategy use is a powerful predictor of general learning behaviors.

### 1.4. Mediation of Self-Efficacy between Metacognitive Strategy, General Learning Behaviors, and Cognitive Strategies

The research results obtained on the effects that metacognitive strategies have on the motivational aspect, as discussed above, have been aligned with the finding by Bandura and Schunk, which showed that students that were given proximal goals were more efficacious than students that were provided with distal goals [17]. Thus, we can predict that there is a process in which metacognitive strategies promote self-efficacy, and in turn self-efficacy enhances general learning behaviors.

However, it is still unclear whether metacognitive strategies do, in fact, enhance self-efficacy. Therefore, it would be useful to obtain empirical support for the theoretical framework pertaining to motivational regulation. Moreover, factors related to motivation, such as self-efficacy, are thought to enhance deep cognitive processing [18,19]. Thus, we predict that self-efficacy mediates the relationship between metacognitive strategy use and the use of deep-processing strategies, as well as general learning behaviors. In addition, we assume that there could be a reciprocal process in which self-efficacy fosters the use of metacognitive strategies [5]. This would imply the need for a methodological solution, so as to examine the causal relationship between self-efficacy and metacognitive strategies.

### 1.5. Purpose of the Current Study

Previous research has not typically considered mediating factors in examining how various elements pertaining to self-regulated learning relate to each other. Thus, the purpose of this study was to examine the effects of the use of metacognitive strategies on self-regulated learning processes, focusing on the mediating effects of self-efficacy. First, we examine the relationships between these two strategies. We predict that planning strategy use promotes monitoring strategy use, based on the framework of the phase of self-regulated learning [1].

**Figure 1:**
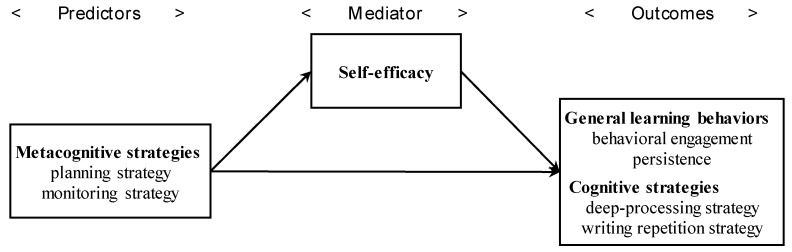
Hypothesized model of the current study.

Second, we investigate the relationships between the two metacognitive strategies, general learning behaviors, and cognitive strategies, focusing on the mediating role of self-efficacy. The hypothesized model is explained below (Figure 1). We predict that the two metacognitive strategies enhance both general learning behaviors and cognitive strategy use. In particular, the use of both metacognitive strategies promotes general learning behaviors via self-efficacy, while monitoring strategy use directly enhances cognitive strategy use.

Preceding the examination of the mediating role of self-efficacy between metacognitive strategies and learning outcomes (i.e., general learning behaviors and cognitive strategies), we also examine the causal relationships between metacognitive strategies and self-efficacy. This is because past research suggested the existence of reciprocal relationships between motivational components and self-regulation [20]. In order to accomplish this purpose, we utilized cross-lagged effect structural equation modeling [21], which allows the causality to be determined based on longitudinal data.

## 2. Materials and Methods

### 2.1. Participants and Procedures

The surveys were conducted in December 2016 (Time 1) and January 2017 (Time 2). The participants were 105 Japanese undergraduates (42 males) enrolled at a comprehensive university in Japan, with an average age of 19.77 (SD = 0.84). All of them took the class on personality psychology and completed the questionnaires during the class in both sessions. The face sheet provided instructions for the survey’s completion, and it also informed all participants to avoid cooperating on the survey and that they could skip items which they found difficult to answer.

The current study was based on the same data source as that of Akamatsu, Nakaya, and Koizumi’s study [22]. We used different measures from their study, with the exceptions of metacognitive strategies and deep-processing strategy use. Moreover, our scope is different from that of Akamatsu et al.’s study, in that we aimed to examine the functions of metacognitive strategies, while Akamatsu et al. investigated reciprocal relationships between effective learning strategies and learners’ beliefs about learning.

### 2.2. Measures

We conducted surveys by using paper-based questionnaires that included the following measures:

Metacognitive strategies: We used items of the metacognitive scale by Umemoto [11] and Sato [10], comprising “planning strategy” (three items; e.g., “I study along the plan that I made”) and “monitoring strategy” (three items; e.g., “I study checking whether I remember what I learned”).

Self-efficacy: We used the self-efficacy scale by Yamaguchi [23]. It was comprised of six items (e.g., “I think that I can understand what is taught during the class”). Yamaguchi reported that Cronbach’s alpha coefficient was 0.89.

Persistence in learning: We used the lack-of-persistence scale [11,24]. It comprised six items (e.g., “I get tired of studying very quickly”). Umemoto reported that Cronbach’s alpha coefficient was 0.78 [11].

Behavioral Engagement: We used the behavioral engagement scale by Umemoto and Tanaka [24], which is the Japanese version of Skinner, Kindermann, and Furrer’s scale [25]. The scale comprised four items (e.g., “I pay attention in class”). Umemoto and Tanaka reported that Cronbach’s alpha coefficient was 0.81.

Cognitive strategies: We used the subscales of “deep-processing strategy” (six items; e.g., “When studying, I link new content in my mind to what I have learned”) and “writing-repetition strategy” (three items; e.g., “When memorizing words, I study by repeatedly writing them down”) of Umemoto’s cognitive strategies scale [11]. Umemoto reported that Cronbach’s alpha coefficient was 0.70 for deep-processing strategy and 0.80 for writing-repetition strategy.

For each scale, responses were placed on a seven-point scale ranging from “not true at all (1)” to “very true (7)”.

## 3. Results

### 3.1. Confirmatory Factor Analysis

Data were analyzed by utilizing the R (version 3.5.1), which is an open-source statistical software package. The lavaan package [26], designed in part to provide the tool to perform latent variable modeling for R users, was utilized to compute the confirmatory factor analysis. In the analyses of this study, we applied the full-information maximum likelihood method, dealing with missing values, assuming MAR (Missing at Random).

The results of the confirmatory factor analyses showed that fit indices of each scale were acceptable (GFI ≧ 0.97, AGFI ≥ 0.97 CFI ≧ 0.90, RMSEA ≦ 0.13, SRMR ≦ 0.08). Further, we computed ω coefficients for each item group in the T1 and T2 data. Although the ω coefficients of the monitoring strategy were low (α = 0.55 in Time 1, α = 0.54 in Time 2), the coefficients of the other variables were judged as acceptable (α = ≧0.81). Scale scores were then computed by calculating the average of each item.

### 3.2. Structure Equation Modeling

First, we performed cross-lagged structure equation modeling, to examine the causal relationships between metacognitive strategies and self-efficacy (Table 1). Fit indices showed good model fit (GFI = 1.00, AGFI = 1.00 CFI = 1.00, RMSEA = 0.00, SRMR = 0.00). It revealed that all autoregressive paths were significant (b* = 0.48–0.76, *p* < 0.01). Furthermore, it showed paths from planning strategy to monitoring strategy (b* = 0.23, *p* < 0.01) and self-efficacy (b* = 0.14, *p* < 0.05).

Second, a path analysis (maximum likelihood method) was performed, in order to examine the process by which metacognitive strategies determine general learning behaviors and cognitive strategies mediated by self-efficacy. We used metacognitive strategies in Time 1 and self-efficacy in Time 2. In constructing the model, we first set paths from the planning strategy to the monitoring strategy and self-efficacy, considering the causality shown in cross-lagged structural equation modeling. We then set direct paths from metacognitive strategies to general learning behaviors and cognitive strategies, in addition to mediated paths via self-efficacy, in order to examine both (a) the mediating effects of self-efficacy and (b) the direct effects without it. In order to examine the mediation effects, we set the path from the monitoring strategy to self-efficacy though cross-lagged structural equation modeling and did not detect the path. Furthermore, we assumed correlations between errors of each learning outcome. As a result, fit indices of the final model indicated an adequate model fit (GFI = 1.00, AGFI = 1.00 CFI = 1.00, RMSEA = 0.00, and SRMR = 0.00).

**Figure 2:**
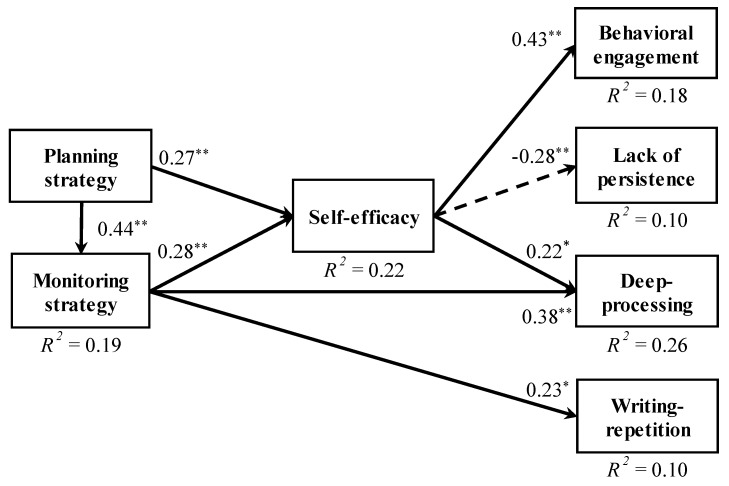
Results of path analysis (N = 102). Insignificant paths and correlations between errors are not depicted in this figure. (* *p* < 0.05, ** *p* < 0.01).

Figure 2 shows the results of the analysis. The coefficients of determination (*R^2^*) ranged from 0.10 to 0.26. Between metacognitive strategies and self-efficacy, positive paths were significant from the planning strategy (b* = 0.27, *p* < 0.01) and monitoring strategy (b* = 0.28, *p* < 0.01) to self-efficacy. From self-efficacy, the positive path to behavioral engagement (b* = 0.43, *p* < 0.01) and the negative path to a lack of persistence (b* = 28, *p* < 0.01) were significant. Moreover, the path from self-efficacy to the deep-processing strategy was significant (b* = 22, *p* < 0.05). Furthermore, direct positive paths from monitoring to the deep-processing strategy (b* = 0.38, *p* < 0.01) and writing-repetition strategy (b* = 0.23, *p* < 0.05) were significant. On the other hand, immediate paths from metacognitive strategies to general learning behaviors were not significant.

Finally, we performed mediation analyses [27,28], in order to examine whether self-efficacy mediates between metacognitive strategies and learning outcomes (general learning behaviors and cognitive strategy use). We used all the variables except for writing-repetition strategy in the path analysis, as the variables showed significant relationships mediated by self-efficacy. We calculated 95 percent confidence intervals of the mediation effects based on a bootstrap sampling method. First, in the mediation analyses on relationships between planning strategy and learning outcomes, each 95 percent confidential interval of indirect effects did not include zero (*b* = 0.12 (0.05, 0.21) on behavioral engagement; *b* = −0.07 (−0.16, −0.01) on lack of persistence; and *b* = 0.08 (0.03, 0.14) on deep-processing strategy), while each direct effect was not significant, indicating the complete mediation effects of self-efficacy. Second, in the analyses on relationships between the monitoring strategy and the learning outcomes, the 95 percent confidential interval of indirect effects did not include zero (*b* = 0.19 (0.10, 0.34) on behavioral engagement; *b* = −0.12 (−0.29, −0.03) on lack of persistence; and *b* = 0.07 (0.01, 0.17) on deep-processing strategy). Direct effects were not significant between the monitoring strategy, behavioral engagement, and lack of persistence. On the other hand, the effect between the monitoring strategy and deep-processing strategy were significant (*b* = 0.32, *p* < 0.01), indicating the partial mediation effect of self-efficacy.

## 4. Discussion

The theoretical framework of self-regulated learning has evolved and established a great deal of empirical evidence in understanding how students autonomously regulate their learning. In particular, past research has long claimed the importance of metacognitive strategies, both in cognitive and motivational aspects. Our objective was to examine the effects of two metacognitive strategies (i.e., planning strategy and monitoring strategy), focusing on the mediating effects of self-efficacy.

### 4.1. Relationships between Self-Efficacy and Metacognitive Strategies

Prior to the examination of the mediating effects of self-efficacy, we investigated the relationships between self-efficacy and metacognitive strategies by performing cross-lagged effect structural equation modeling. The analysis revealed causal relationships from planning strategy to monitoring strategy. Past research proposed that self-regulated learning has the following three successive phases; forethought, performance, and reflection [1]. Planning strategy is closely related to the first phase, while monitoring strategy is related to the second phase. Considering that the first phase precedes the second, the causal relationship from planning strategy to monitoring strategy is perhaps intuitively sound.

Next, the analysis showed another causal relationship whereby the planning strategy promotes self-efficacy. Students can gain self-efficacy by setting clear or proximal goals. This result supports the previous studies on motivational regulation strategies, demonstrating the effect of metacognitive strategies on learner motivation [13,16].

On the other hand, the analysis did not find the causal relationship that the monitoring strategy enhanced self-efficacy. This result seems to contradict the findings that proximal goal setting includes reflection on performance. We can infer that not many students gain self-efficacy merely by applying the monitoring strategy, as low achievers could not be efficacious by simply monitoring their low performance. However, this claim should be limited, as we tested causality under strict conditions to detect the effect such, as small sample size and controlling for prior self-efficacy.

In contrast to the effects of the planning strategy, no causal relationships from self-efficacy to metacognitive strategies were identified. Previous research, based largely on correlational data, has conceptualized self-efficacy as a significant predictor of self-regulated learning strategies that include metacognitive strategies [5,29]. Thus, the current results are curious, as they ostensibly contradict the theoretical framework in which self-efficacy plays a notable role in predicting the use of self-regulated learning strategies. Further research is warranted here, so as to examine and establish the causal relationship between self-efficacy and self-regulated strategy use, perhaps by involving larger sample sizes.

### 4.2. Mediating Effects of Self-Efficacy between Metacognitive Strategies, General Learning Behaviors, and Cognitive Strategies

A path analysis and mediation analyses indicated important relationships between metacognitive strategy use, general learning behaviors, and cognitive strategy use. First, metacognitive strategies enhanced behavioral engagement and persistence, mediated by self-efficacy. Past research has long shown the relationships between metacognitive strategies and general learning behaviors [13,14,15,16]. However, the mechanism through which the strategy use promotes general learning behaviors was not sufficiently understood. This study located self-efficacy as a mediating factor between them and revealed a complete mediating effect. This indicates that metacognitive strategy use leads to general learning behaviors by inducing self-efficacy. This process aligns with the assumption of the research on motivational regulation [13,16].

In contrast, regarding the relationships between metacognitive strategies and cognitive strategies, it was shown that monitoring-strategy use directly promoted the use of deep-processing and writing-repetition strategies. This result accords with the results in Sato’s study [10] and the theoretical framework of metacognition [4]. As the monitoring strategy entails learners’ reflections of their own understanding and learning strategies, students who use the strategy are presumably more conscious of their cognitive processes and, hence, tend to apply cognitive strategies more.

In addition, self-efficacy somewhat mediated between metacognitive strategy use and deep-processing strategy use. This result demonstrates that self-efficacy, induced by planning or monitoring, deepens learning processing. Although the indirect effect was relatively small, it appears to be sufficiently meaningful and calls for further investigation. Deep cognitive strategy use is determined by self-efficacy apart from the directive and cognitive process arising from monitoring strategy, as higher cognitive processing is also induced by high motivation [18]. Taking the fact that this mediation did not exist between the monitoring strategy and the writing-repetition strategy, we can infer that self-efficacy induces deeper cognitive processing rather than surface processing [1].

### 4.3. Implications for Educational Practice

This study revealed several functions of two metacognitive strategies. This finding provides some educational implications, particularly showing how teachers should support students with different academic problems, such as motivational and cognitive problems. Because students’ metacognitive strategy use can be enhanced by supporting learning environments, such as teachers and technological systems [30,31], we can provide solutions to these problems by intervening the two metacognitive strategies. The first is students who do not seem behaviorally engaged or who lack persistence; this is presumably caused by a low level of self-efficacy. For such students, by instructing them how to apply the planning strategy (e.g., set proximal goals and study according to a plan), teachers can improve their self-efficacy, which in turn promotes students’ general learning behaviors.

Second, students can experience difficulties in knowing how to study or learn something, even though they are behaviorally engaged. This can be attributed to a lack of cognitive strategies. For such students, we can encourage their active use of cognitive strategies by instructing them in the monitoring strategy, as it directly determines cognitive strategy use. This accessibility to improve students’ learning is enabled by the differentiation of the functions of the two metacognitive strategies.

### 4.4. Limitations

This study examined the processes though which metacognitive strategies determine learning outcomes, and it shed some light on the crucial mediating role played by self-efficacy. However, the study has several methodological and theoretical limitations. First, our sample size of 105 students might not have been sufficient for performing a complex set of analyses pertaining to a multitude of factors. Second, we were unable to find the causality whereby self-efficacy determined the use of metacognitive strategies; therefore, assuming reciprocal relationships between them seems to be more valid. Third, we used self-efficacy in the context of learning in general. Individuals have differing self-efficacy, both qualitatively and quantitatively, along different domains, and previous research has shown that self-efficacy is more meaningful when conceptualized specifically in terms of the tasks at hand. We therefore need to consider self-efficacy along various domains, such as the academic domain and specific tasks, and to examine whether they have the same mediating effects. Taken together, future research is required to address these problems, which will allow us to elaborate on the model proposed in this study. Ultimately, refining the model presented in this study will allow researchers and educators to deepen our understanding of the intertwined relationships among the use of various learning strategies, self-efficacy, and learning behaviors, which, in turn, will allow them to devise intervention strategies to promote learning.

### 4.5. Conclusions

In this article, we investigated the processes though which metacognitive strategies determine learning outcomes. The results of our analyses showed the meaningful mediation of self-efficacy. The findings can refine the theoretical framework of SRL that has long claimed importance of self-efficacy and metacognitive strategies. Also, the results propose that we can enhance students’ learning by interventions focusing on metacognitive strategy use and self-efficacy. More future research is required to extend and validate these ideas gained from our examinations.

## Figures and Tables

**Table 1:** Results of cross-lagged effect model.

Correlations between Exogenous Variables		Regressions		*R* ^2^
Plan (Time 1)—Moni (Time 1)	0.43 **	Plan (Time 1)→Plan (Time 2)	0.71 **	0.48
Plan (Time 1)—SE (Time 1)	0.32 **	Moni (Time 1)→Plan (Time 2)	−0.08	
SE (Time 1)—Moni (Time 1)	0.45 **	SE (Time 1)→Plan (Time 2)	0.06	
**Correlations between Errors**		Plan (Time 1)→Moni (Time 2)	0.23 **	0.38
Plan (Time 2)—Moni (Time 2)	0.35 **	Moni (Time 1)→Moni (Time 2)	0.48 **	
Plan (Time 2)—SE (Time 2)	0.05	SE (Time 1)→Moni (Time 2)	0.00	
SE (Time 2)—Moni (Time 2)	0.30 **	Plan (Time 1)→SE (Time 2)	0.14 *	0.66
		Moni (Time 1)→SE (Time 2)	−0.01	
		SE (Time 1)→SE (Time 2)	0.76 **	

(1) Plan: Planning strategy; Moni: Monitoring strategy; SE: Self-efficacy; (2) * *p* < 0.05, ** *p* < 0.01.
